# Biosynthesis of Macrocyclic Peptides with C-Terminal β-Amino-α-keto Acid Groups by Three Different Metalloenzymes

**DOI:** 10.1021/acscentsci.4c00088

**Published:** 2024-04-11

**Authors:** Dinh T. Nguyen, Lingyang Zhu, Danielle L. Gray, Toby J. Woods, Chandrashekhar Padhi, Kristen M. Flatt, Douglas A. Mitchell, Wilfred A. van der Donk

**Affiliations:** ^†^Department of Chemistry, ^‡^Carl R. Woese Institute for Genomic Biology, University of Illinois at Urbana-Champaign, Urbana, Illinois 61801, United States; § School of Chemical Sciences NMR Laboratory, University of Illinois at Urbana-Champaign, Urbana, Illinois 61801, United States; ∥ School of Chemical Sciences George L. Clark X-Ray Facility and 3M Materials Laboratory, University of Illinois at Urbana-Champaign, Urbana, Illinois 61801, United States; ⊥ Materials Research Laboratory, University of Illinois at Urbana-Champaign, Urbana, Illinois 61801, United States

## Abstract

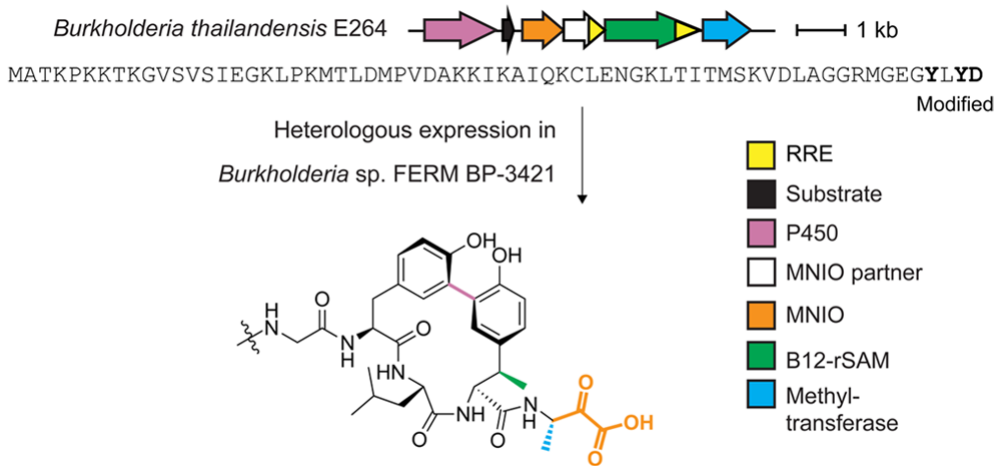

Advances in genome sequencing and bioinformatics methods have identified a myriad of biosynthetic gene clusters (BGCs) encoding uncharacterized molecules. By mining genomes for BGCs containing a prevalent peptide-binding domain used for the biosynthesis of ribosomally synthesized and post-translationally modified peptides (RiPPs), we uncovered a new compound class involving modifications installed by a cytochrome P450, a multinuclear iron-dependent non-heme oxidative enzyme (MNIO, formerly DUF692), a cobalamin- and radical *S*-adenosyl-l-methionine-dependent enzyme (B12-rSAM), and a methyltransferase. All enzymes were functionally expressed in *Burkholderia* sp. FERM BP-3421. Structural characterization demonstrated that the P450 enzyme catalyzed the formation of a biaryl C–C cross-link between two Tyr residues with the B12-rSAM generating β-methyltyrosine. The MNIO transformed a C-terminal Asp residue into aminopyruvic acid, while the methyltransferase acted on the β-carbon of this α-keto acid. Exciton-coupled circular dichroism spectroscopy and microcrystal electron diffraction (MicroED) were used to elucidate the stereochemical configuration of the atropisomer formed upon biaryl cross-linking. To the best of our knowledge, the MNIO featured in this pathway is the first to modify a residue other than Cys. This study underscores the utility of genome mining to isolate new macrocyclic RiPPs biosynthesized via previously undiscovered enzyme chemistry.

## Abstract 2

Three metalloenzymes and a methyltransferase convert a ribosomally translated peptide into an atropisomeric biaryl macrocyclic peptide with a C-terminal β-amino-α-keto acid and two *C*-methylations.

## Introduction

Natural products are prolific sources of structurally diverse and biologically active compounds of high societal value.^1^ The rapid expansion of genomic sequence databases, combined with the development of high-throughput, accurate, and open-access bioinformatics tools, has unveiled many new natural product biosynthetic pathways.^2−5^ Just as advances in directed evolution revolutionized the use of enzymes in commodity chemical synthesis, enzymes sourced from natural product biosynthetic pathways have become a rich reservoir for the development of innovative biocatalytic processes.^6−9^

Ribosomally synthesized and post-translationally modified peptides (RiPPs) are a large and growing family of natural products featuring a diverse range of molecular scaffolds.^10^ The ribosomal precursor peptide frequently contains an N-terminal leader region typically responsible for recruiting the modifying enzymes, while a C-terminal core region receives the post-translational modifications.^11^ Exceptional transformations catalyzed by RiPP enzymes result in diverse structural moieties^10^ possessing antimicrobial, antiviral, antifungal, herbicidal, cytotoxic, anticancer, and other activities.^12−14^ With nearly 50 reported structural classes, defined by the post-translational modification(s) (PTMs) installed, RiPP biosynthesis is a highly productive arena for the discovery of new enzyme chemistry,^15−29^ identification of versatile bioengineering catalysts,^30−33^ and isolation of structurally exotic compounds with unprecedented biological modes of action.^34,35^ While tools like AlphaFold have provided high-quality structures for millions of enzymes,^36^ a major unsolved challenge is the determination of enzyme function. Typically, one does not know the substrate for a novel enzyme of interest.^37^ However, this challenge is simplified for enzymes involved in prokaryotic RiPP biosynthesis, as the substrate(s) are typically encoded near the modifying enzyme(s) in a biosynthetic gene cluster (BGC).^10^ Advances in identifying and analyzing short open-reading frames in prokaryotic genomes enabled reliable annotation of RiPP precursor peptides of known classes and high-confidence prediction of precursor peptides of yet-uncharacterized RiPP classes.^4,38−40^

Our approach to finding BGCs that encode first-in-class RiPPs starts with bioinformatic searches centered on a prevalent class-agnostic protein domain termed the RiPP precursor recognition element (RRE).^39,41,42^ These domains are found in the majority of prokaryotic RiPP BGCs but have been difficult to identify, owing to their small size (80–90 residues), frequent fusion to much larger proteins, and high sequence variability. All known RRE domains contain three alpha helices and a three-stranded beta sheet. The cognate precursor peptides are engaged as the fourth strand of the beta sheet, often with nanomolar affinities. While the structural fold of RREs is well-conserved, sequence homology tools such as BLASTP are insufficient to retrieve RRE domains across RiPP classes. This diversity has necessitated the use of hidden Markov model (HMM)-based retrieval for cataloging RRE domains. The tool RRE-Finder uses a collection of custom HMMs and HHpred-based remote homology detection methods to identify these domains,^42^ which serve as an incomplete but useful biomarker of a RiPP BGC. This allows the prioritization of RiPP BGCs that encode novel collections of enzymes or hypothetical enzymes with unknown functions. The uniqueness of the precursor peptide is also considered paramount in BGC prioritization, as it is the foundation on which the final RiPP is constructed.^4,43^ This procedure was recently followed to discover a new class of RiPPs, now termed the daptides, which modify an invariant C-terminal Thr into (*S*)-*N*_2_,*N*_2_-dimethyl-1,2-propanediamine.^39^ Thus, daptides are ribosomal peptides with two N-termini.

In this work, we pursued the structural characterization of a RiPP BGC from *Burkholderia thailandensis* E264 that is unlike any other reported. The BGC uniquely encodes three metalloenzymes that could transform the precursor peptide into an unprecedented scaffold. Indeed, we show the cumulative actions of a multinuclear non-heme iron-dependent oxidative enzyme (from protein family PF05114, abbreviated as MNIO), a cobalamin- and radical *S*-adenosyl-l-methionine-dependent enzyme, a cytochrome P450, and a methyltransferase. The cytochrome P450 installed a biaryl macrocyclic peptide formed from two Tyr residues, with one Tyr also undergoing *C*-methylation on an unactivated sp^3^ carbon center catalyzed by the B12-rSAM. Subsequent catalysis by the MNIO on the resulting biaryl atropisomer-containing peptide converted the C-terminal Asp residue to aminopyruvic acid. Given that the MNIO is fully conserved in these RiPP BGCs, we consider it to be class-defining. We therefore termed the products aminopyruvatides and named the BGCs that produce them as *apy*. We also demonstrate that *Burkholderia* sp. FERM BP-3421 is a superior heterologous host superior to *Escherichia coli* for some of the PTMs in this pathway. Overall, this study suggests that a combination of RRE-centric genome mining and reconstitution of biosynthetic pathways in heterologous hosts beyond *E. coli* offers advantages for exploring the natural product chemical space.

## Results

### Bioinformatic Survey and Experimental Target Selection

**Figure 1:**
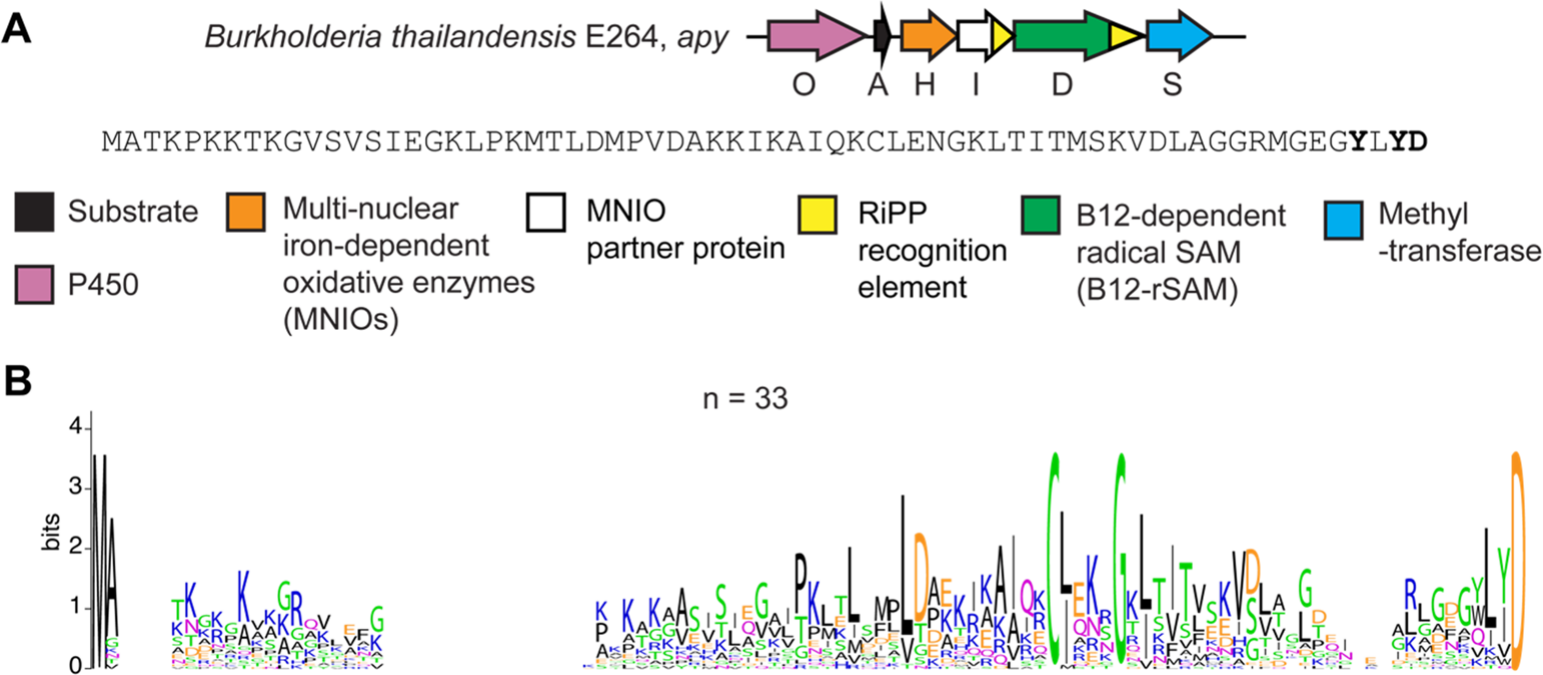
Overview of the *apy* cluster and conserved amino acid motifs in predicted precursor peptides from similar clusters. (A) BGC diagram of the RiPP pathway of interest (*apy*) from *B. thailandensis* E264 and the sequence of the precursor peptide. Amino acid residues that undergo modifications are bolded. (B) Sequence logo of ApyA precursor peptides from homologous BGCs with identical sequences removed (unique sequences = 33, total sequences = 69).

We sought out an RRE-dependent RiPP BGC encoding multiple uncharacterized metalloenzymes, given their unparalleled ability to catalyze difficult and diverse chemical transformations.^44−46^ A BGC obtained from RRE-Finder that caught our interest was from *B. thailandensis* E264 and is composed of a sequence-unique substrate peptide (ApyA, NCBI accession code: ABC34935.1), an MNIO enzyme (ApyH, ABC35712.1),^26^ an RRE-containing protein (ApyI, ABC34269.1), a methyltransferase (ApyS, ABC34725.1), an RRE-containing cobalamin- and radical *S*-adenosyl-l-methionine-dependent enzyme (B12-rSAM, ApyD, ABC34219.1),^46,47^ and a cytochrome P450 enzyme (ApyO, ABC35200.1) (Figure 1 and supplementary data set 1).^45,48^ To obtain homologous BGCs, we performed a BLASTP search of each enzyme against the nonredundant NCBI protein database. The genome neighborhood and putative precursor peptides corresponding to the collected homologues were obtained using RODEO,^4^ and BGCs encoding putative precursor(s) were analyzed. During this process, we noticed that the precursor peptides were sequence-diverse with only a central CBX_2–3_G motif (X = any amino acid, B = hydrophobic amino acid) and a C-terminal Asp as the conserved features. This knowledge was then utilized to identify probable precursor peptides. This workflow identified highly similar BGCs that were prevalent in the *Burkholderia pseudomallei* group. Select strains from other Pseudomonadota and the phylogenetically distant Actinomycetota also contain putative *apy* BGCs (supplementary data set 1). The homologous BGCs contain an MNIO (100% occurrence), a B12-rSAM (97% occurrence), and a methyltransferase (93% occurrence), whereas only 74% contains the P450 enzyme. A sequence alignment of the predicted precursor peptides highlighted the previously mentioned conserved C-terminal Asp and CBX_2–3_G motif, which a priori could not be assigned as being part of the leader or core region (Figure 1B). Further, we noticed that two aromatic (either Trp or Tyr) residues were only present as the second and fourth residue from the C-terminus when the BGC encoded a P450 enzyme. Identification of such correlations are often informative for determining the site(s) of enzymatic action.

### Assignment of Enzymatic Activities via Heterologous Expression in *E. coli*

Initially, we constructed an *E. coli* expression plasmid encoding the precursor peptide (ApyA) containing an N-terminal hexa-His tag and all associated modifying enzymes in a pRSF-based vector (Table S1). All genes were codon-optimized for *E. coli* expression. Given the reliance of the B12-rSAM (ApyD) on cobalamin and the inability of *E. coli* to synthesize vitamin B12 and derivatives, we co-transformed a pCDF-based vector containing the vitamin B12-uptake pathway (*btu*) and supplemented the growth medium with cobalamin.^49,50^ The peptide products resulting from co-expression were purified using Ni-NTA affinity chromatography, digested with endoproteinase LysC or GluC, and then subjected to high-resolution electrospray ionization mass spectrometry (HR-ESI-MS).

**Figure 2:**
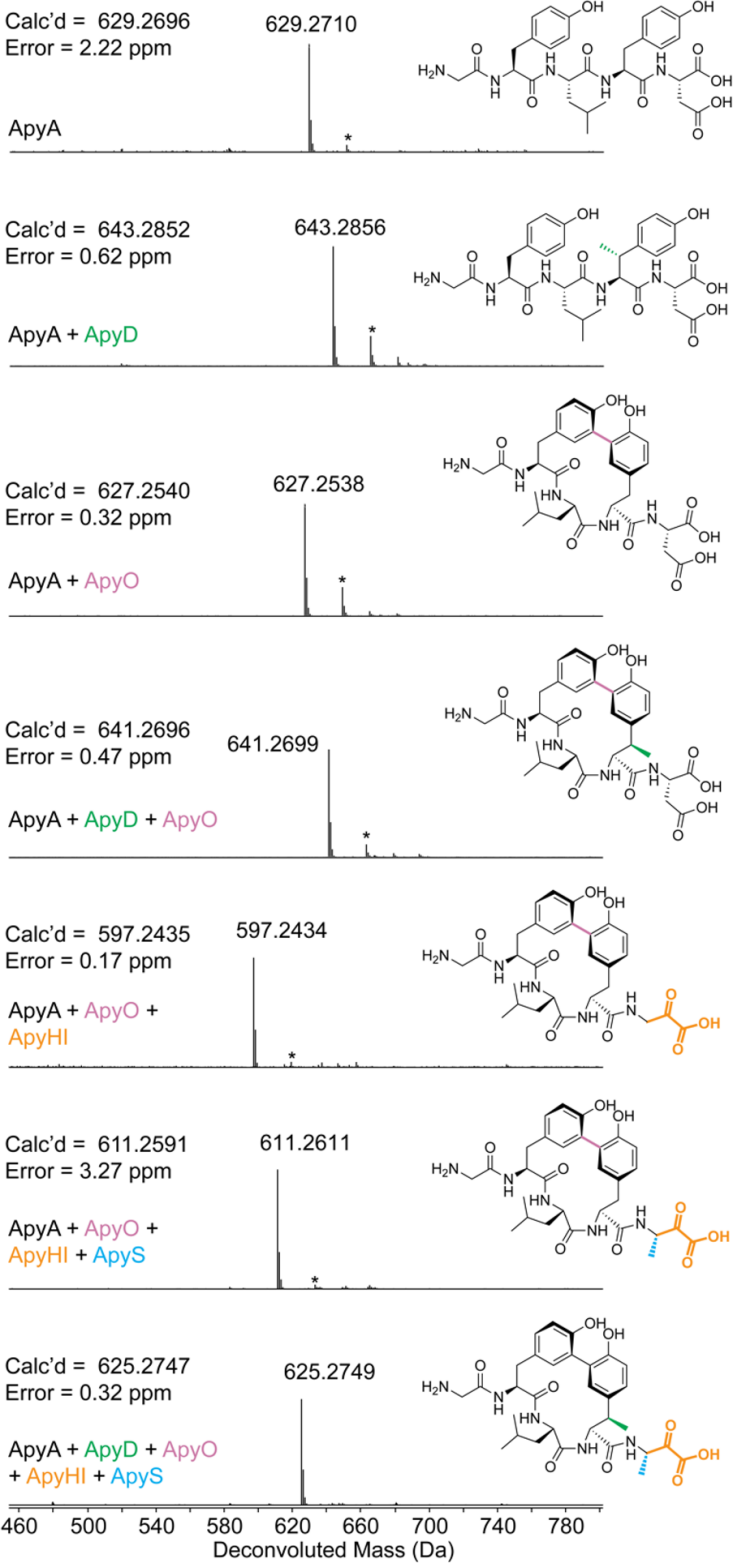
Deconvoluted HR-ESI mass spectra and elucidated structures of ApyA modified by different combinations of enzymes followed by proteolysis with GluC. Structures drawn in the second, third, fourth, and sixth spectra were verified by 2D-NMR spectroscopy and stereochemical analysis (Marfey’s method or exciton-coupled circular dichroism). The structure drawn in the third spectrum was also verified by MicroED. Other drawn structures were inferred from HR-ESI-MS/MS experiments and determined enzyme functions. An asterisk (*) denotes [M + Na]^+^. ApyA, precursor peptide; ApyD (green), B12-rSAM; ApyO (pink), cytochrome P450; ApyHI (orange), MNIO and partner protein; and ApyS (blue), methyltransferase. The top four spectra were from expression in *E. coli*, and the bottom three spectra were from expression in *Burkholderia* sp. FERM BP-3421. All products were purified by HPLC prior to MS analysis.

**Figure 3:**
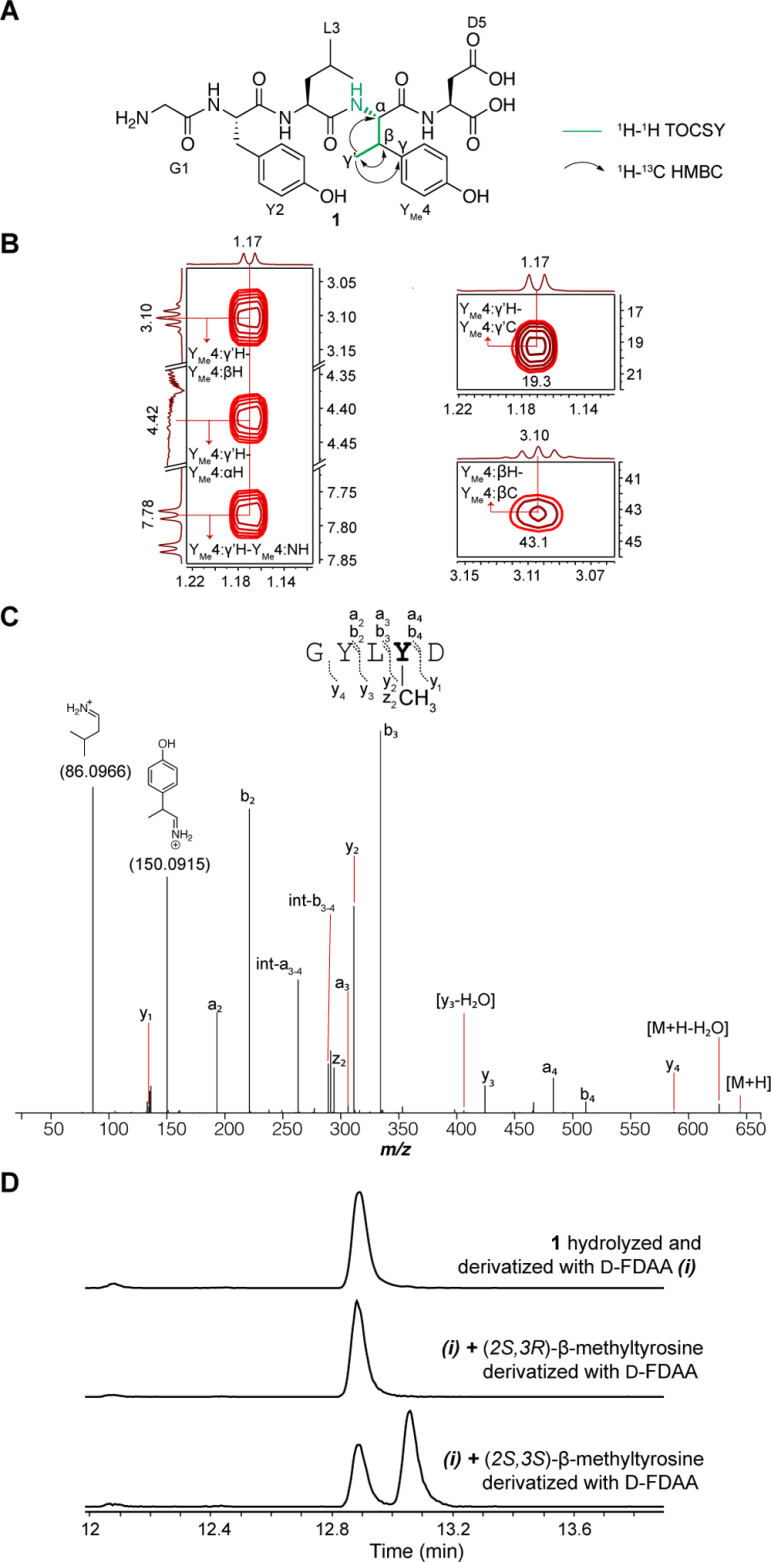
Structural elucidation of the ApyD-catalyzed modification. (A) Key NMR correlations consistent with the presence of β-methyltyrosine in peptide **1** (axes in ppm). (B) NMR spectral data of diagnostic cross peaks indicating the position of the new methyl group. Left: ^1^H–^1^H TOCSY. Right: ^1^H–^13^C HSQC. The ^1^H–^13^C HMBC key correlations can be found in Figure S17F. (C) HR-ESI-MS/MS data showing the site of methylation. int = internal fragment. A mass table with observed error can be found in supplementary data set 3. (D) Extracted ion chromatograms (EICs, *m*/*z* = 700.1937) of **1** or authentic standards after hydrolysis and derivatization with Marfey’s reagent (d-FDAA). The EICs of **1** and authentic standards after derivatization with l-FDAA are shown in Figure S18.

Upon co-expression of ApyA and the rSAM ApyD and *btu*, an increase of +14 Da was observed, suggestive of a single methylation (Figures 2 and S1). HR-ESI tandem mass spectrometry (HR-ESI-MS/MS) localized the mass increase to the penultimate residue of ApyA (Tyr), which is found in a Gly-Tyr-Leu-Tyr-Asp motif (Figure 3). Upon co-expression of ApyA with the cytochrome P450 ApyO, a mass loss of 2 Da was observed (Figures 2 and S1). HR-ESI-MS/MS analysis localized this change to the Tyr-Leu-Tyr region, and the lack of observable single fragmentation in this part of the peptide was suggestive of a cross-link (Figure S2). Co-expression of ApyA with ApyO and ApyD/*btu* yielded a mass change of +12 Da, consistent with the ApyO-catalyzed 2 Da mass loss and ApyD-catalyzed 14 Da mass gain (Figure S1). Co-expression of ApyA with ApyHI (MNIO and RRE domains) in *E. coli* did not lead to any mass changes. Co-expression of ApyA with all protein components encoded by the BGC (ApyODHIS) yielded the same mass deviations observed upon co-expression with just ApyO and ApyD (Figure S1). These results imply that ApyHI and ApyS were either nonfunctional in our *E. coli* experiments or performed mass-neutral modifications that preserved the molecular formula. As described below, we were able to characterize the function of these latter enzymes in a different heterologous host.

### Assignment of Enzymatic Activities via Heterologous Expression in *Burkholderia*

We assigned the functions of ApyHI and ApyS by performing heterologous expression in *Burkholderia* sp. FERM BP-3421. This emerging chassis strain is closely related to *B. thailandensis* E264 and has been shown to significantly enhance the production of RiPP and non-RiPP natural products.^51−53^ To facilitate expression, we inserted the genomic DNA containing the *apy* BGC downstream of the rhamnose-inducible promoter in a pSCrhaB2 plasmid used previously for protein expression in *Burkholderia* (Table S2).^54^ Additionally, we introduced an N-terminal hexa-His tag on ApyA to facilitate isolation. Upon co-expression of the entire *apy* BGC and Ni-NTA purification, the major species observed was the ApyA precursor peptide with a mass loss of 18 Da (Figures 2 and S3). Analysis of tandem MS data suggested that a loss of 2 Da localized to the Tyr-Leu-Tyr region was identical to the ApyO-catalyzed modification observed in *E. coli* (Figure S4). The remaining loss of 16 Da was confined to the conserved C-terminal Asp residue (Figure S4). Omission of ApyH or ApyI from the co-expression cultures resulted in no modification of the C-terminal Asp (Figures S5–S8). Omission of ApyS from the expression trials resulted in a 32 Da mass loss from unmodified ApyA (Figure S9). Given the 2 Da mass loss assigned to the P450 reaction, the remaining 30 Da mass loss was attributed to ApyHI. Tandem MS localized this mass deviation (30 Da) to the C-terminal Asp residue (Figure S10). Collectively, these data suggested that ApyHI is active upon expression in *Burkholderia* but not in *E. coli* and works together to modify the C-terminal Asp. This finding was unexpected since all reported reactions for MNIOs modify Cys residues.^16,26,55^ Furthermore, these data suggest that ApyS catalyzes a methylation reaction on the ApyHI-modified C-terminal Asp residue.

Our initial expression trials in *Burkholderia* sp. FERM BP-3421 appeared to produce active ApyHI, ApyO, and ApyS, resulting in an ApyA product that decreased in mass by 18 Da. We also observed a minor species with a mass 14 Da larger than the M–18 Da product (i.e., 4 Da decreased from unmodified ApyA; Figure S3), suggesting low efficiency of ApyD-catalyzed methylation. Cobalamin supplementation to the growth medium and expression optimization improved the methylation status, although the reaction remained incomplete (Figures S11–S13). To improve the ApyD-catalyzed methylation, we redesigned the expression vector (Figure S14 and Table S3) to introduce a separate ribosome-binding site for *apyD* instead of utilizing the native gene architecture with overlapping *apyH*, *apyI*, and *apyD* genes. This construct resulted in the ApyA/O/HI/D/S product (Figure 2) being the major product (Figures S11 and S15), as supported by HR-ESI-MS/MS data (Figure S16). The mass loss of 4 Da compared to unmodified ApyA was accounted for by a putative Tyr-Tyr cross-link catalyzed by ApyO (−2 Da), methylation of the penultimate Tyr by ApyD (+14 Da), an unknown (−30 Da) modification to the C-terminal Asp catalyzed by ApyHI, and methylation by ApyS (+14 Da).

### Structural Elucidation of the ApyD and ApyO Reaction Products

The MS/MS data described thus far suggested that ApyD methylates the penultimate Tyr of the ApyA peptide; however, the precise site of methylation remained unclear. We therefore prepared a larger quantity of ApyD-modified ApyA in *E. coli*, digested the product with endoproteinase GluC, and purified the resulting methylated Gly-Tyr-Leu-Tyr-Asp pentapeptide by high-performance liquid chromatography (HPLC). Multidimensional nuclear magnetic resonance (NMR) spectroscopy was then used to elucidate the structure (Figures 3 and S17). A combination of ^1^H–^1^H total correlation spectroscopy (TOCSY) and ^1^H–^1^H rotating-frame Overhauser effect spectroscopy (ROESY) was used to assign the ^1^H chemical shifts (Table S4). Importantly, the ^1^H–^1^H TOCSY spectrum showed a spin system containing N–H, C_α_–H, C_β_–H, and the new methyl group (γ′). ^1^H–^13^C heteronuclear single quantum coherence (HSQC) analysis demonstrated the β-carbon of the Tyr to be CH instead of CH_2_, and ^1^H–^13^C heteronuclear multiple bond correlation (HMBC) analysis produced correlations between the new methyl group, C_β_–H, and C_α_–H of Tyr and the C4 carbon of the aromatic side chain (Figure S17F). Overall, these spectroscopic data support the ApyD-catalyzed formation of β-methyltyrosine.

To ascertain the stereochemical configuration of the β-methyltyrosine product, Marfey’s method was employed.^56,57^ Specifically, the methylated pentapeptide was hydrolyzed using 6 M DCl/D_2_O, followed by derivatization with both the l- and d-forms of Marfey’s reagent (1-fluoro-2-4-dinitrophenyl-5-alanine amide) (Figure S18). The commercial synthetic standards (2*S*,3*R*)-β-methyltyrosine and (2*S*,3*S*)-β-methyltyrosine were subjected to analogous hydrolysis and derivatization conditions. Co-elution in LCMS of the derivatized β-methyltyrosine obtained enzymatically with the similarly derivatized authentic standard of (2*S*,3*R*)-β-methyltyrosine (Figures 3 and S18) allowed for the assignment of the ApyD-catalyzed product as (2*S*,3*R*)-β-methyltyrosine.

We next determined the structure of ApyO-modified ApyA by utilizing a similar workflow. Integration of the ^1^H NMR spectrum of the pentapeptide detected only six aromatic C–H protons instead of the eight that would be expected for two unmodified Tyr residues. This finding suggested a C–C biaryl cross-link (Figure S19 and Table S5). The spin system of the aromatic rings observed by ^1^H and ^1^H–^1^H TOCSY showed two groups of peaks with splitting patterns of doublet (d, 8 Hz), d of doublets (dd, 8 Hz, 2 Hz), and d (2 Hz) (Figure S19G). This change in the aromatic spin system (compared to unmodified Tyr) along with correlations between the *meta*-hydrogen (d, 2 Hz) of ring 1 and the *ortho*-carbon of ring 2 (and vice versa, demonstrated by ^1^H–^13^C HMBC), supported a cross-link between the *ortho*-carbons of each Tyr residue (Figure S19J). Analogous 2D-NMR experiments on the peptide resulting from the co-expression of ApyA with both ApyO and ApyD were consistent with the product containing the C–C cross-link and β-methyltyrosine (Figure S20 and Table S6).

We next assigned the stereochemistry of the atropisomer formed by the ApyO-catalyzed biaryl cross-link. We utilized exciton-coupled circular dichroism (ECD), a non-empirical method for the assignment of the spatial orientation between chromophores absorbing at similar wavelengths.^58^ We first obtained ECD spectra of four chiral synthetic molecules with a similar aromatic C–C cross-link. The ECD spectra of molecules with *R* axial chirality exhibited a negative first and positive second Cotton effect, while the ECD spectra of molecules with *S* axial chirality exhibited the opposite Cotton effect (Figure S21). Analysis of the ApyO-modified peptide yielded a negative first (307 nm) and positive second Cotton effect (285 nm), suggesting a counterclockwise orientation between the two Tyr residues, and hence, we assigned *R* axial chirality (Figure S22). Control samples lacking the biaryl cross-link did not exhibit these ECD signals (Figure S23).

**Figure 4:**
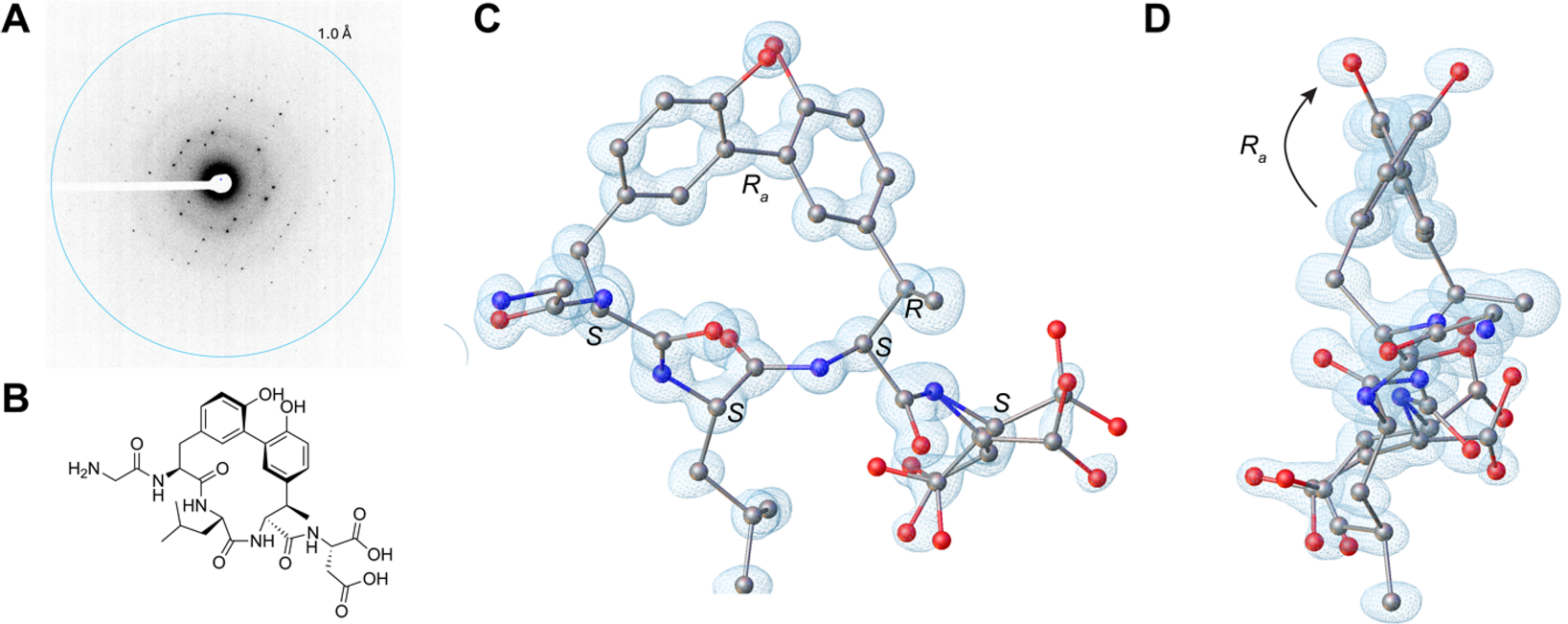
Determination of the ApyD- and ApyO-modified ApyA pentapeptide structure by MicroED. (A) Diffraction pattern of the peptide crystal with resolution rings set at 1.0 Å. (B) Drawing of the chemical structure of ApyD- and ApyO-modified ApyA pentapeptide. (C) MicroED structure of ApyD- and ApyO-modified ApyA pentapeptide at 1.0 Å resolution. CCDC ID 2324739. The blue mesh represents the observed electron density map (*F*_obs_). The full structure comprises two complete peptide molecules with a Zn atom and water molecules (see Figure S24 and the Supplementary CIF data). Here, only one peptide molecule is shown, and hydrogen atoms are not shown for clarity. (D) Side view of the structure to highlight the *R* axial chirality.

We next investigated whether the ApyD- and ApyO-modified ApyA product exhibited similar atropisomerism as the ApyO-modified ApyA product, as modifications on the macrocycle can result in changes in the atropisomeric state.^59,60^ ECD spectra of the GluC-cleaved ApyA/D/O product exhibited a significant positive Cotton effect like the ApyA/O product but no negative Cotton effect (Figure S23). This lack of observable exciton coupling has been noted on other chiral molecules.^61,62^ We then turned to the cryo-electron microscopy (cryo-EM) method of microcrystal electron diffraction (MicroED).^63−67^ The diffraction data from two nanocrystals were merged to yield a nearly complete data set with 1.0 Å resolution (Figure 4). The structure of the ApyA/D/O product was then determined by direct methods, which demonstrated *R* axial chirality for the biaryl ring similar to the ApyA/O product, suggesting that the β-methylation of the Tyr residue did not alter the prevailing atropisomer (Figures 4 and S24 and Table S7). Furthermore, the assignment by Marfey’s method of the 2*S*,3*R* configuration of β-methyltyrosine was confirmed by MicroED. The structure was deposited under CCDC ID 2324739.

### Structural Elucidation of the ApyO/HI/S Reaction Product

**Figure 5:**
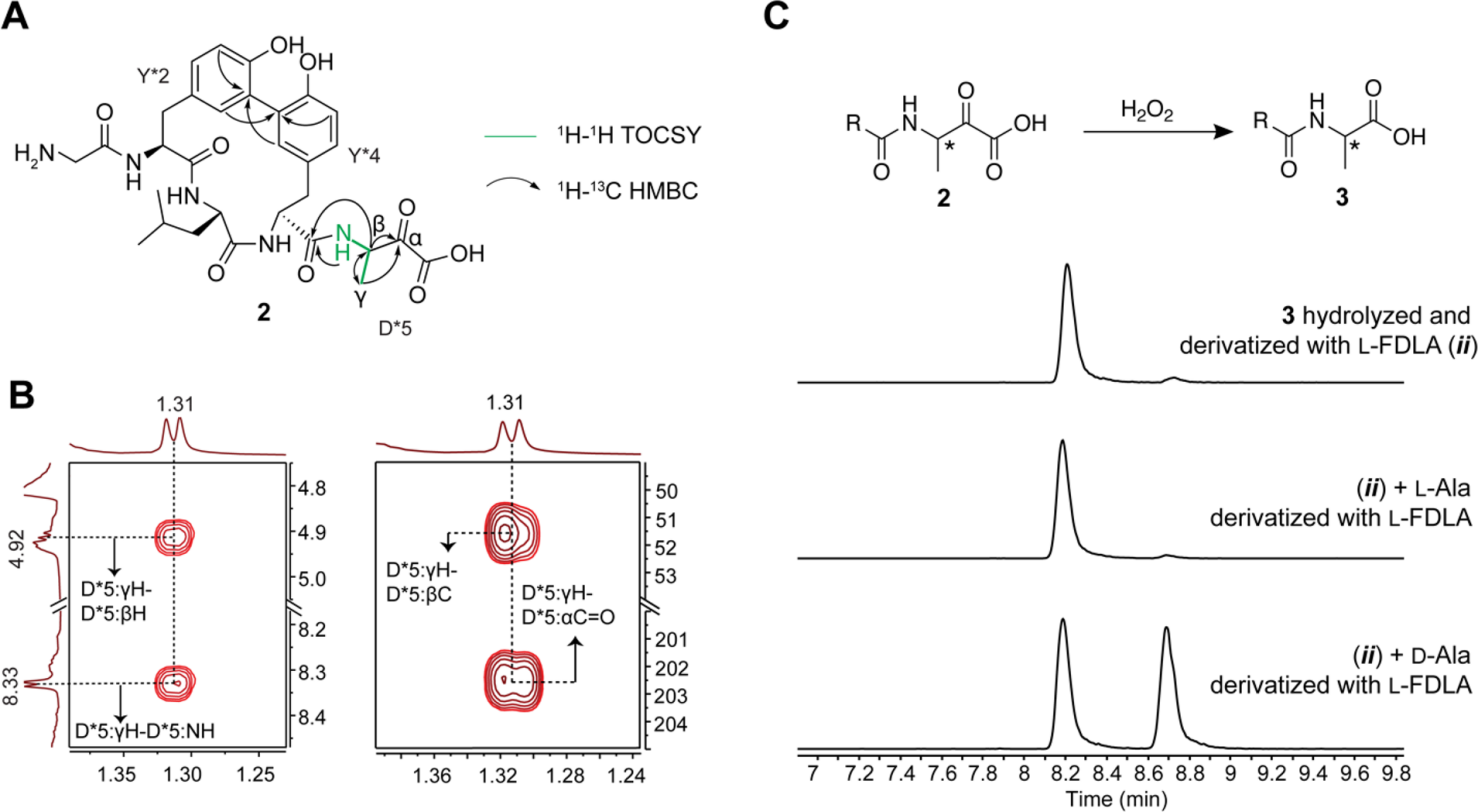
Structural elucidation of the chemistry performed by ApyO, ApyHI and ApyS. (A) Depiction of the 2D-NMR data consistent with a C–C biaryl cross-link and C-terminal 3-amino-2-oxobutanoic acid in peptide **2**. (B) 2D-NMR spectra (left: ^1^H–^13^C HMBC; right: ^1^H–^1^H TOCSY) with key cross peaks characteristic of the C-terminal 3-amino-2-oxobutanoic acid. Other key cross peaks can be found in Figure S27I. (C) Schematic depiction of the oxidation of the C-terminal 3-amino-2-oxobutanoic acid by H_2_O_2_ to an Ala residue, and the EIC (*m*/*z* = 382.1368) of hydrolyzed and derivatized Ala residues after Marfey’s derivatization.

We next determined the structures of the ApyA modification by ApyHI and ApyS. Omission of ApyO from co-expression resulted in the absence of a modification of the C-terminal Asp, suggesting that the biaryl cross-link is a prerequisite for ApyHI and/or ApyS activity (Figures S25 and S26). Consequently, we prepared a larger quantity of ApyA modified by ApyO, ApyHI, and ApyS in *Burkholderia* sp. FERM BP-3421 for structure determination (Figure S27 and Table S8). Analysis of the pentapeptide product after GluC proteolysis confirmed the C–C cross-link between the *ortho*-carbons of the two Tyr (Figures 5 and S27G,H and Table S8). ^1^H–^1^H TOCSY and ^1^H–^13^C HSQC analyses revealed a new spin system for the structure originating from the C-terminal Asp that was composed of a N–H amide (8.35 ppm), a C–H group with chemical shifts reminiscent of α-positions of amino acid residues (4.91 ppm for ^1^H and 51.6 ppm for ^13^C), and a new methyl group (1.31 ppm) (Figure 5 and S27B,E,I). The ^1^H–^13^C HMBC spectrum revealed correlations between the carbon atoms in the C-terminal structure and all the C–H protons and a new carbonyl based on the ^13^C chemical shift (202.5 ppm) (Figure S27I). A secondary species was detected containing a N–H amide (7.81 ppm), a C–H group (4.18 ppm for ^1^H and 50.5 ppm for ^13^C), and a methyl group (1.04 ppm) on the C-terminal residue (Figure S27J,K). The ^1^H–^13^C HMBC cross peak between the methyl protons and a carbon exhibiting a chemical shift consistent with a geminal diol (95.1 ppm) suggested this secondary species represented the hydrate of the new ketone, as the data were acquired in 10% D_2_O and 90% H_2_O. Owing to this equilibrium, during the mixing period in the pulse sequences of the ^1^H–^1^H nuclear Overhauser effect spectroscopy (NOESY) and ^1^H–^1^H ROESY experiments, the signal for the γ-methyl group of the ketone (1.31 ppm) exchanged to the chemical environment of the γ-methyl group of the geminal diol (1.04 ppm), represented by a cross peak connecting the two frequencies observed at the end of the pulse sequences (Figure S27L). This exchange was further supported by this cross peak between the two methyl groups having the same phase as the diagonal peaks in the ^1^H–^1^H ROESY spectrum, whereas all other ROE cross peaks arising from spatial proximity between two nuclei had the opposite phase to the diagonal peaks (Figure S27D,L).^68^ These results, combined with the HR-ESI-MS data showing ApyHI catalysis resulted in a loss of 30 Da from unmodified ApyA, suggested that ApyHI and ApyS converted the C-terminal Asp residue to a C-terminal 3-amino-2-oxobutanoic acid (Figure 5).

The stereochemistry at the β-carbon of the newly installed α-keto acid was determined by Marfey’s analysis. To circumvent acid-catalyzed epimerization adjacent to the ketone, we first oxidized the α-keto acid to a carboxylic acid using H_2_O_2_ (Figures 5 and S28).^69,70^ The successful conversion of the C-terminal 3-amino-2-oxobutanoic acid to Ala further affirmed the structural assignments. We then performed acid hydrolysis and derivatization with the advanced Marfey’s reagent 1-fluoro-2,4-dinitrophenyl-5-l-leucine amide and, in parallel, derivatized authentic standards of l- and d-Ala.^71^ LC-MS analysis indicated that the derivatized Ala post peptide hydrolysis was l-configured, suggesting that the new C-terminus installed by ApyHI and ApyS was (*S*)-3-amino-2-oxobutanoic acid.

As previously stated, omission of ApyS from co-expression experiments abolished methyl group transfer to the C-terminus of the modified peptide, implying ApyHI converted the C-terminal Asp residue into aminopyruvic acid (Figures 2, S9, and S10). Supporting this hypothesis, we obtained an AlphaFold^36^ model of ApyS, and the closest structural homologue identified by DALI^72^ was MppJ, a methyltransferase converting phenylpyruvic acid to 3-methylphenylpyruvic acid (Figure S29).^73,74^ The structural and functional resemblance supports the finding that ApyS *C*-methylates the relatively nucleophilic β-carbon of the α-keto acid generated by ApyHI.

### The Conserved Cys of ApyA Is Essential for MNIO Activity

ApyHI is the first MNIO-RRE pair to catalyze the modification of a non-Cys residue, leading us to question whether the centrally located, conserved CBX_2–3_G motif of ApyA was left unmodified (Figure 1B). HR-ESI-MS/MS data indicated that no other segment of ApyA besides the C-terminal Tyr-Leu-Tyr-Asp motif was enzymatically modified in mass (Figures S30 and S31). To examine the possibility that ApyHI performed an isobaric modification to the central Cys residue (Figure 1), we reacted ApyHI-treated ApyA with iodoacetamide, which yielded an *S*-alkylated product, indicating the presence of a sulfhydryl group. The peptide was then digested with endoproteinase GluC and trypsin, generating an *S*-alkylated Cys-Leu-Glu tripeptide for examination by NMR spectroscopy (Figure S32 and Table S9) and Marfey’s analysis (Figures S33 and S34). The ^1^H–^13^C HSQC spectrum of the alkylated peptide revealed two CH_2_ moieties, one from the β-carbon of Cys and one from the carbamidomethyl group, along with a CH moiety from the α-carbon of Cys (Figure S32E). In addition, we observed a ^1^H–^13^C HMBC cross peak between protons on the CH_2_ carbamidomethyl group and the β-carbon of Cys. Concurrently, the ^1^H–^1^H TOCSY spectrum revealed a spin system consistent with that of unmodified Cys. This conclusion was further bolstered by Marfey’s analysis of the *S*-alkylated tripeptide (Figures S33 and S34). Authentic standards of l-Cys and d-Cys were reacted with iodoacetamide and Marfey’s reagent. The alkylated Cys residue in ApyHI-modified ApyA after acid hydrolysis and Marfey’s derivatization co-eluted with the derivatized l-Cys standard, confirming that the conserved Cys residue was indeed unmodified.

To investigate any possible role of the conserved Cys on ApyA in supporting the activity of ApyHI on the C-terminal Asp, we substituted the Cys with Ser, Ala, Asp, and His. In each case, the activity of ApyHI was abolished, underscoring the importance of the conserved Cys (Figures S35–S37). Replacement of the Gly in the CBX_2–3_G motif with Ala led to a reduced modification efficiency, while Val substitution resulted in no detectable ApyA modification. Thus, while the conserved CBX_2–3_G motif is not chemically modified in the product, it is important for ApyHI activity on ApyA. These substitutions in ApyA did not affect ApyO activity, as we still observed the P450-cyclized product.

## Discussion

Using the class-agnostic RRE domain to identify BGCs encoding an unprecedented arrangement of metalloenzymes, we identified an intriguing case from *B. thailandensis* E264. Similar BGCs were found in Pseudomonadota and Actinomycetota. Among the encoded metalloenzymes, the selected BGC encodes ApyD, a B12-dependent rSAM enzyme. Other members of this family have been reported to perform methylation, C–S bond formation, C–P bond formation, ring contraction, and C–C cyclization.^75^ To the best of our knowledge, ApyD is the first enzyme reported to methylate the Cβ of Tyr. In contrast, ApyO catalyzes biaryl cross-linking between two Tyr residues, a reaction that has been observed with the non-RiPP natural products arylomycin (AryC)^76^ and mycocyclosin (CYP121),^77^ albeit with different substrates and stereochemical outcomes.^78,79^ C–C bond formations involving two aromatic rings have been reported to be catalyzed by cytochrome P450 enzymes in RiPP biosynthesis,^45,48,80,81^ with a recent example (SlyP) catalyzing a similar cross-link as ApyO but without information on atropisomerism.^80^ Some of the precursor peptides in the identified BGCs here have other aromatic residues in the second and fourth positions from the C-terminus (Trp/Tyr; see supplementary data set 1), suggesting that other biaryl linkages will be formed. Since P450s are not fully conserved among the BGCs we identified, the RiPPs described here are not classifiable as biarylitides.^21,82^

Perhaps the most interesting modification observed was catalyzed by ApyHI (a member of the multinuclear non-heme iron-dependent oxidative enzyme superfamily, MNIO), which are universally conserved among *apy* BGCs and are thus considered class-defining. Previously characterized MNIOs catalyze distinct chemical reactions exclusively on Cys, although a very recent report describes an MNIO catalyzed N–Cα bond cleavage of an Asn to generate a C-terminal amide in a methanobactin-like pathway.^83^ ApyHI instead performs an unprecedented enzymatic modification on a C-terminal Asp. A conserved Cys in the precursor peptide appears critical for ApyHI, with substitution by other amino acids abolishing enzyme activity; currently, we cannot distinguish whether the Cys is involved in catalysis or in substrate binding/orientation.

We propose a mechanism for the MNIO-catalyzed reaction that first involves a four-electron oxidation of the β-carbon to form a ketone (Figure S38), consistent with the four-electron oxidations catalyzed by all previously characterized MNIOs.^16,26,55^ This mechanism involves hydroxylation at the β-carbon, followed by radical formation at the same carbon. This radical intermediate may undergo proton-coupled electron transfer to form a ketone, and the resulting β-keto acid could then undergo decarboxylation to yield the observed C-terminal aminopyruvic acid. Alternatively, the radical intermediate could decarboxylate^84−87^ to form an enol that would tautomerize and form the α-keto acid product. Further work is necessary to substantiate the proposed enzymatic mechanisms for ApyHI.

The ApyHI product is an oxidized analogue of β-alanine, and after *C*-methylation by ApyS, an analogue of β-homoalanine is formed. Hence, ApyHI provides a route to β-amino acids containing a ketone functionality at the C-terminus of a ribosomally synthesized peptide, akin to the function of spliceases, rSAM enzymes that form such structures within a peptide.^15^ Given that the spliceotide products are protease inhibitors,^88^ the final product of the *apy* BGC may function in a similar manner.^89^ At present, it is unclear whether modified ApyA will be further processed by a leader peptidase. The BGC does not encode a protease, but akin to other RiPPs, the protease could be encoded elsewhere on the genome.^90^ Attempts to elicit the production of the final product by *B. thailandensis* E264 to provide information on proteolytic processing were unsuccessful.

It is common to utilize *E. coli* as a heterologous host when characterizing RiPP BGCs.^91^ While the use of *E. coli* remains highly successful, it is an insufficient vessel to obtain functional enzymes from every desired biosynthetic pathway.^92−94^ Indeed, although we did not investigate the reason, ApyHI was not active in *E. coli* under the conditions employed. Instead, we demonstrated here the successful utilization of a *Burkholderia* host to obtain active biosynthetic enzymes from a novel *Burkholderia*-derived BGC. This example illustrates the value of looking beyond *E. coli* for future RiPP characterization.^95−97^ This study also showcases the discovery of new enzyme functions by mining for RiPP BGCs, which contain information on both substrate(s) and biosynthetic enzymes.

**Figure 6:**
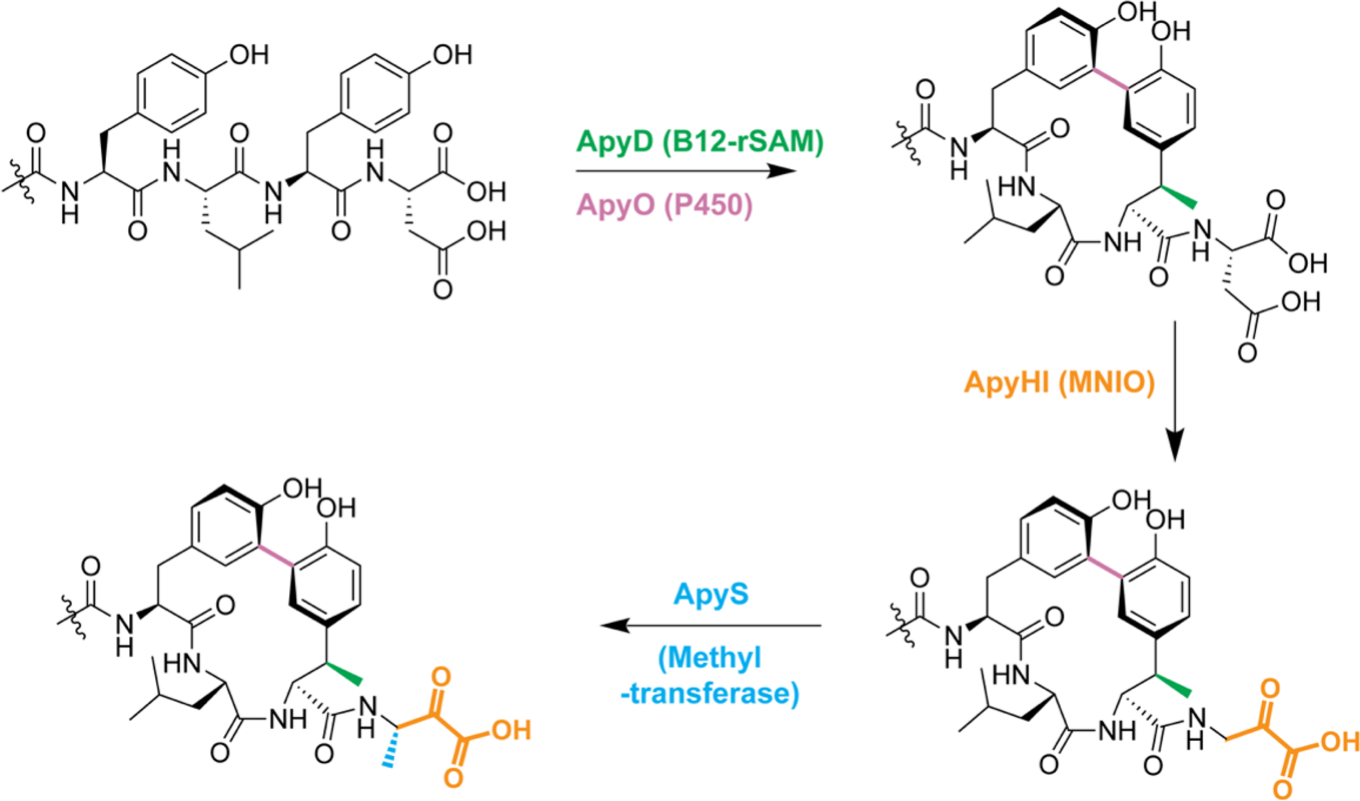
Proposed biosynthetic pathway for the *apy* BGC from *B. thailandensis* E264. B12-rSAM: cobalamin- and radical *S*-adenosyl-l-methionine-dependent enzyme. P450: cytochrome P450 enzyme. MNIO: multinuclear non-heme iron-dependent oxidative enzyme.

In conclusion, we report the bioinformatic discovery and experimental characterization of a novel RiPP class with modifications coming from three distinct families of metalloenzymes. We functionally expressed each enzyme and elucidated the structures of the resulting products by HR-ESI-MS/MS, 2D-NMR, ECD, Marfey’s analysis, and MicroED. Our data suggest that the activity of ApyS is dependent on the α-keto acid moiety installed by ApyHI. Furthermore, the removal of ApyO resulted in the abolishment of the modifications catalyzed by ApyHI and ApyS. We thus suggest the post-translational pathway to start with the installation of the (2*S*,3*R*)-β-methyltyrosine by the B12-dependent rSAM enzyme ApyD and *R*_a_ biaryl cross-link formation by the cytochrome P450 ApyO. These steps are apparently independent of each other. Next, the C-terminal Asp residue is converted to aminopyruvic acid by the MNIO ApyHI. Lastly, methylation of the β-carbon of the aminopyruvic acid by the methyltransferase ApyS forms a C-terminal (*S*)-3-amino-2-oxobutanoic acid (Figure 6).

## Supporting Information Available

Experimental procedures, Figures S1–S38, and Tables S1–S9 showing spectroscopic data and gene sequences (PDF)
Supplementary data set 1: bioinformatic output (ZIP)
Supplementary data set 2: nucleotide sequences (XLSX)
Supplementary data set 3: mass spectral data (XLS)
Supplementary CIF data: MicroED structural data in CIF format (CIF)

## Supplementary Material

oc4c00088_si_004.xls

oc4c00088_si_005.cif
